# Agency Penalties From Taking Parental Leave for Women in Men-Dominated Occupations: Archival and Experimental Evidence

**DOI:** 10.1007/s11199-024-01517-7

**Published:** 2024-09-11

**Authors:** Ivona Hideg, Anja Krstić, Raymond Nam Cam Trau, Yujie Zhan, Tanya Zarina

**Affiliations:** 1 Saïd Business School, University of Oxford, Park End St, Oxford, OX1 1HP UK; 2 Schulich School of Business, York University, 111 Ian Macdonald Boulevard, Toronto, ON M3J 1P3 Canada; 3 Faculty of Liberal Arts & Professional Studies, York University, 4700 Keele Street, Toronto, ON M3J 1P3 Canada; 4 Macquarie Business School Macquarie University, North Ryde, NSW 2109 Australia; 5 Lazaridis School of Business & Economics, Wilfrid Laurier University, 75 University Avenue West, Waterloo, ON N2L 3C5 Canada; 6 Toronto, Canada

**Keywords:** Gender equality, Working women, Stereotyped attitudes, Employees leave benefits, Men-dominated occupations, Parental leaves, Gender stereotypes

## Abstract

Organizations have started more progressively using and offering family benefits including parental leaves to address the issues of balancing work and family life. Although such leaves are fundamental for supporting, attracting, and retaining women, we examine whether such leaves may also inadvertently affect women’s careers in occupations that overly value masculine traits, unless managed carefully. Drawing on the literature on gender stereotypes (micro factors) and occupation gender type (macro factors), we argue that longer (vs. shorter) parental leaves negatively affect women’s work outcomes (i.e., annual income, salary recommendation, hireability, and leadership effectiveness) in men-dominated but not in women-dominated occupations because it lowers perceptions of women’s agency. We find support for our hypotheses across three studies in the Australian context with an archival data set and two experiments. Our work shows that men-dominated organizational structures reinforce traditional gender stereotypes, whereas such reinforcement does not happen in women-dominated organizational structures. Our research equips leaders and organizations with insights into the unintended negative consequences of parental leave for women. This understanding serves as a crucial first step in developing strategies and programs to mitigate these effects, thereby supporting women in men-dominated occupations and fostering more inclusive and healthy workplaces.

Women continue to face barriers in entering and succeeding in traditionally men-dominated occupations; they represent only 34% of the workforce in science and engineering occupations in the United States, and 23.5% and 14% in Canada and Australia (Catalyst, 2022). One of the key findings identified in past literature is that women face prominent challenges in men-dominated occupations stemming from the challenge of balancing work and family life (Ceci & Williams, 2011). Although challenges to achieving work-family balance are experienced in many occupations, men-dominated occupations in particular tend to be inconsistent with work-family balance given highly agentic and masculine cultures where long hours and high demands are the norm (e.g., Cech & Blair-Loy, 2019; Frome et al., 2008; Shauman, 2017). In response to these challenges, organizations in men-dominated industries have started offering more family-friendly policies, such as parental leave benefits, to attract and retain women. For example, men-dominated industries in the United States, such as finance and technology, seem to offer the most generous parental leaves compared to other industries (Werber, 2017). However, these leaves are more often offered to women, and women are also more encouraged to take them, compared to men (Heymann, 2016).

Although access to parental leave is necessary for women’s health and success at work in general (Hideg et al., 2018; Staehelin et al., 2007), the effect of the length of parental leave on women’s work outcomes in men-dominated occupations is less understood. Understanding this effect is critical as longer parental leave may emphasize women’s traditional gender roles, which may conflict with agentic and masculine demands of men-dominated occupations, undermining women’s success at work. Indeed, past research suggests that parental leave may have unintended negative effects on women’s careers (e.g., Evertsson, 2016; Lequien, 2012; Olivetti & Petrongolo, 2017). As such, a key question is whether parental leave may hinder women’s success at work, thus reinforcing, rather than solving, gender inequities.

In this paper, we consider how parental leave length impacts women’s work outcomes in men-dominated occupations by integrating micro and macro factors undermining gender equity. We draw on and integrate the literature on gender stereotypes and social role theory (Eagly & Wood, 2012; Koenig & Eagly, 2014) and structural barriers (i.e., occupation gender type; Joshi et al., 2015; Reskin, 1993) to suggest that women who take longer parental leave incur negative work-related outcomes (e.g., pay and promotion) in men-dominated occupations, but they do not in women-dominated occupations. A longer parental leave (even though it may be a standard length of legislated leave under a particular policy) may highlight women’s caregiving role, which is at odds with agentic traits (i.e., dedication to work and ambition) that are seen as necessary for career success and general fit in traditionally men-dominated occupations (Cejka & Eagly, 1999; Deutz et al., 2022). As such, undermined perceptions of agency stand to decrease the perceived value women bring to their workplaces where a premium is placed on such a gendered trait. By contrast, in women-dominated occupations where agency is not seen as paramount, parental leaves may not be related to adverse outcomes for women.

We test our predictions that women in men-dominated occupations would experience negative work outcomes when they take longer versus shorter parental leaves (an effect not observed in women-dominated occupations) in the context of Australian parental leave policies. In Study 1, we establish external validity by using an archival data set to examine the relation between women’s parental leave length and their annual post-leave income in men-dominated, women-dominated, and gender-neutral occupations. In Study 2, we provide internal validity for our results by replicating and extending Study 1 findings in an experiment using a sample of Australian workers and comparing women who took a standard length of parental leave (12 months) versus a one-month parental leave. In another experiment in Study 3, we examine undermined perceptions of agency as a mediator of the negative effect of longer parental leave incurred by women in men-dominated occupations.

## Theory Development

Past research has documented unintended negative effects of parental leave on women’s work-related outcomes including reduced wages (Evertsson, 2016), lower perceptions of commitment and hireability (Hideg et al., 2018), and lower access to advancement (Olivetti & Petrongolo, 2017). It is notable that these negative effects have also been observed in countries with more progressive and longer parental leave policies such as Sweden (Evertsson, 2016; Evertsson & Duvander, 2011), Germany (Fitzenberger et al., 2016), and Canada (Hideg et al., 2018). However, this past work has not considered the effect of occupation gender type (the characteristic of an occupation regarding whether and how it is dominated by one gender) in escalating (or potentially ameliorating) negative effects of parental leaves for women’s careers. Examining the boundary effect of occupation gender type is critical as women are particularly underrepresented in men-dominated occupations, yet men-dominated occupations may accentuate traditional gender stereotypes bringing on unintended negative effects of parental leave for women. Below, we present our theorizing on how women’s agency is undermined in men-dominated occupations when they take longer parental leaves.

## Parental Leave and Agency Stereotypes in Men-Dominated Occupations

A large body of research on gender stereotypes posits that women are perceived as highly communal (e.g., oriented towards others, warm, compassionate), whereas men are thought to be highly agentic (e.g., oriented towards the self, ambitious, assertive; Bakan, 1966; Diekman & Eagly, 2000; Eagly et al., 2020). According to social role theory (Eagly & Wood, 2012; Koenig & Eagly, 2014), these gender stereotypes stem from the social roles that women and men occupy in society. Women traditionally occupy caregiver roles which require nurturing and communal traits, which has led to the inference that women are especially communal. Similarly, men traditionally occupy breadwinner roles which emphasize achievement and authority, which has led to the inferences that men are especially agentic (Cejka & Eagly, 1999). The stereotypes of women being communal (and not very agentic) and men being agentic (and not very communal) have changed little over time (Eagly et al., 2020).

In line with these gender stereotypes, research suggests that women have difficulties entering men-dominated professions due to a perceived lack of fit between women being stereotyped as communal and occupations that are perceived as requiring agentic traits for success (Eagly & Karau, 2002; Heilman, 2001; Heilman et al., 2015). Further, women occupying men-dominated positions need to demonstrate agentic traits and/or be perceived as agentic to ‘fit in’ and advance their careers. Indeed, past research shows that women who occupy men-dominated occupations are seen as agentic (e.g., assertive and ambitious; Bosak et al., 2012; Froehlich et al., 2020). Yet, given the persistent gender stereotypes that women are communal, such increases in women’s agency perceptions in men-dominated fields can be fragile. A highly salient communal event, namely taking a parental leave, may undermine agency perceptions. Specifically, taking a longer parental leave may be seen at odds with expectations of high agency in men-dominated occupations (Joshi et al., 2015). That is, occupation gender type signals what kind of behaviors are normative and desirable and what kind of behaviors may violate standard expectations and may be penalized (Joshi et al., 2015; Reskin, 1993). Thus, the longer the parental leave, the more likely women are to be viewed as less ambitious and dedicated to their work, resulting in lower agency perceptions (e.g., Hideg et al., 2018).

Past research shows that perceptions of employees’ agency are consequential for work-related outcomes. Specifically, the more employees are perceived to be dedicated and ambitious, the more they are perceived to contribute to their workplaces and thus should be better rewarded (Cuddy et al., 2011). Given agentic traits are viewed as especially important in men-dominated occupations (Deutz et al., 2022), we expect women working in such fields to experience worsened work-related outcomes (e.g., less pay and promotion opportunities) when they take a longer parental leave, despite being entitled to them, compared to a shorter parental leave.

Further, we expect that taking a longer (vs. shorter) parental leave may be less influential on women’s work-related outcomes in women-dominated occupations. Women-dominated occupations, such as early childhood education and personal care, tend to value relatedness, warmth, and caring (Cejka & Eagly, 1999) as these feminine characteristics can contribute to one’s performance in such occupations. As such, these occupations signal more feminine oriented norms of behavior and taking a parental leave, regardless of its length, may be seen as more normative. As a result, longer parental leaves should not threaten women’s work-related outcomes in women-dominated (vs. men-dominated) occupations. Taken together, we put forward the following hypotheses:

### Hypothesis 1

Parental leave length in men-dominated occupations will be negatively related to women’s work-related outcomes, whereas such negative work-related outcomes will be less likely in women-dominated occupations.

### Hypothesis 2

Undermined perceptions of agency will mediate the negative effect of parental leave length on women’s work-related outcomes in men-dominated occupations.

## Role of Communality

Agency and communality are fundamental dimensions of social cognition (Abele & Wojciszke, 2014) and judgments on one dimension inherently invoke questions about the judgments on the other dimension. Given our theoretical positioning of agency as an underlying mechanism, there is a question of whether perceptions of women’s communality may also act as a mechanism and increase with the length of the parental leave. Communality also conflicts with the demands of traditionally men-dominated occupations; increased communality perceptions may explain the negative impacts of parental leave length on women’s work-related outcomes.

We, however, expect that communality perceptions would not be influenced by the length of parental leave. Women who give birth to or have a child are seen as fulfilling their ultimate feminine role by becoming mothers (Morgenroth & Heilman, 2017). Thus, regardless of the length, parental leave will signal motherhood status, which is related to high levels of communality. We thus expect that regardless of the length of parental leave women take, they will be still perceived by others as similarly high in communality. Further, although agency and communality are considered to be orthogonal constructs, empirically they are often correlated (Abele & Wojciszke, 2014). To empirically address the role of communality and account for the potential correlation between agency and communality, in Study 3 where we examine the mediating mechanism of agency, we also include and assess perceptions of communality in our model as an additional mediator. We do so in an exploratory fashion.

## Study Context, Transparency, and Openness

We test our hypotheses in three studies using a mixed method approach in the context of Australian parental leave policies. Australia has a government mandated parental leave policy which includes being eligible for 52 weeks (one year) of unpaid leave, out of which 22 weeks are paid at the national minimum wage, and an option of requesting an additional one-year unpaid leave (Fair Work Ombudsman, 2024). In Study 1 we used an archival data set, the *Household*,* Income and Labour Dynamics in Australia (HILDA) Survey*, published by the Melbourne Institute. The access and use of this data set are managed and regulated by the Melbourne Institute (please see https://melbourneinstitute.unimelb.edu.au/hilda) and thus we do not have permission to share this data set.

Study 2 and 3 were experiments and were reviewed and approved by an Institutional Review Board. The protocol number was 5142 and the title of the project was “Evaluating job applicants.” Data from Study 2 and 3 cannot be shared publicly because we do not have consent from our participants to do so; however, data are available on an individual basis and can be requested from the first author. For our analyses we used IBM SPSS Statistics 27. We provide all materials for Study 2 and 3 at the following OSF link: https://osf.io/ujacn/?view_only=784379d51d5a47e3a8c9dc1c11553cf0.

## Study 1 Method

### Sample and Procedure

Archival data from the *HILDA Survey* from 2001 to 2013 were used to test Hypothesis 1. This survey was conducted by the Melbourne Institute, which collected information about economic and personal well-being, labor market dynamics, and family life of Australians from a nationally representative sample. The first wave of this survey was conducted in 2001, and the same participants were revisited and have been surveyed every year thereafter. The survey was administered via individual interviews and questionnaires with people aged 15 or older in each household.

For the current study, we selected participants in this survey based on the following criteria. First, participants were women who reported the birth of a child in any wave of data collection and answered the questions about the length of leave before and after the birth of a child (see the measure of parental leave length below for details). For those who reported more than one child birth, we selected the most recent leave or the leave taken for their youngest child. This resulted in an initial sample of 992 participants. Given the outcome variable of interest was post-leave income, we further retained only those participants reporting their income during the second year after returning to work following parental leave, resulting in a sample of 863 participants. We chose to use the salary reported for the second year after returning to work from parental leave because participants returned to work at different times during the financial year and thus some participants did not work for the entire year during the first financial year following a parental leave, threatening the validity of the first financial year income as a measure of annual income.

Finally, given our focus on the role of occupation gender type, we only included participants who answered the item about their occupation during the year before the birth of the child. Thus, our final sample consisted of 657 women (age: *M* = 28.55, *SD* = 5.58) who took parental leave (parental leave length: *M* = 0.94 years, *SD* = 0.89; the year of baby born ranged between 1995 and 2010). The sample included 104 (15.8%) women working in men-dominated occupations, 462 (70.3%) women in women-dominated occupations, and 91 (13.9%) women in gender-neutral occupations (see below for the coding of gender type of occupations). The average gross income in the year before child birth was $42,843.73 Australian dollars (*SD* = 36,584.98) in men-dominated occupations, $32,627.28 in women-dominated occupations (*SD* = 28,776.13), and $27,633.08 in gender-neutral occupations (*SD* = 19,751.53). Average gross income across all occupations was $33,546.90 (*SD* = 29,397.54).

### Measures

#### Work-Related Outcome

We operationalize annual income as a work-related outcome because this information presents a tangible and meaningful work outcome and a frequently examined indicator of gender equity. Specifically, we used a question asking participants to report their financial year gross income in Australian dollars in the second year after returning from parental leave.

#### Parental Leave Length

We used two items to measure this variable. Namely, women were asked to report the length of leave they took before the birth of their child and the length of leave they took after the birth. We calculated a total score by summing the responses to the two items to reflect the total length of parental leave taken. Parental leave length was originally coded in weeks. We rescaled it to number of years in our analysis based on 52 weeks per year.

#### Occupation Gender Type and Pilot Study

The HILDA survey asked participants to report their current occupation and the main tasks and duties in their job. Based on these responses, the researchers of the Melbourne Institute assigned to each participant one of the 51 occupational categories developed by the Australian and New Zealand Standard Classification of Occupations (ANZCO; Australian Bureau of Statistics, 2006). In the process of classifying occupations, ANZCO took into account various information including the skill level and the nature of the tasks in the occupation. We use the two-digit code list of the ANZCO because it gives a sufficient spread of occupational categories for the purpose of constructing a measure of gender composition categories. We coded occupation type based on the results of a pilot study, which we describe below.

In a pilot study we recruited 80 workers in Australia (42 men or 52.5%, and 38 women or 47.5%; 36 employed full-time or 45%, 29 employed part-time or 36.3%, 15 contract or 18.8%; full-time work experience: *M* = 4.53, *SD* = 5.68) to examine participants’ perceptions of the gender composition of 48 occupational categories (please see Table 1 for a full list of all occupational categories) developed by the ANZCO. Participants were presented with 48 occupational categories and were asked to indicate the extent to which each is typically held by men or women using a 7-point Likert scale (1 = *typically held by men*, 4 = *held equally by men and women*, 7 = *typically held by women*) from Brescoll et al. (2012).

**Table 1:** Pilot Study: Means, Standard Deviations, and Results of One-Sample t-Tests Examining the Gender Composition of 48 Occupational Categories

Occupational categories	Mean (standard deviation)	t	Gender composition
1. Managers	3.26 (0.88)	-7.48***	Men-dominated
2. Chief executives, general managers and legislators	2.38 (0.93)	-15.58***	Men-dominated
3. Farmers and farm managers	2.63 (1.08)	-11.35***	Men-dominated
4. Specialist managers	3.56 (0.73)	-5.39***	Men-dominated
5. Hospitality, retail and service managers	4.43 (0.94)	4.05***	Women-dominated
6. Professionals	3.94 (0.37)	-1.52	Gender-neutral
7. Arts and media professionals	4.47 (0.89)	4.80***	Women-dominated
8. Business, human resource and marketing professionals	4.39 (1.16)	2.98**	Women-dominated
9. Design, engineering, science and transport professionals	2.86 (0.95)	-10.70***	Men-dominated
10. Education professionals	4.94 (1.07)	7.83***	Women-dominated
11. Health professionals	4.26 (0.84)	2.80**	Women-dominated
12. ICT professionals	2.58 (1.00)	-12.70***	Men-dominated
13. Legal, social and welfare professionals	4.38 (0.72)	4.67***	Women-dominated
14. Technicians and trades workers	2.24 (1.09)	-14.42***	Men-dominated
15. Engineering, ICT and science technicians	2.84 (1.23)	-11.68***	Men-dominated
16. Automotive and engineering trades workers	1.75 (0.76)	-26.67***	Men-dominated
17. Construction trades workers	1.63 (0.86)	-24.63***	Men-dominated
18. Electrotechnology and telecommunications trades workers	2.54 (1.12)	-11.63***	Men-dominated
19. Food trades workers	4.11 (0.57)	1.75	Gender-neutral
20. Skilled animal and horticultural workers	3.81 (1.05)	1.61	Gender-neutral
21. Community and personal service workers	4.96 (0.95)	9.09***	Women-dominated
22. Health and welfare support workers	5.34 (0.93)	12.91***	Women-dominated
23. Carers and aides	5.36 (0.94)	14.94***	Women-dominated
24. Hospitality workers	4.68 (0.88)	6.73***	Women-dominated
25. Protective service workers	3.45 (1.40)	-3.50**	Men-dominated
26. Sports and personal service workers	3.31 (1.03)	-5.99***	Men-dominated
27. Clerical and administrative workers	4.95 (1.05)	8.06***	Women-dominated
28. Office managers and program administrators	3.94 (1.17)	-0.48	Gender-neutral
29. Personal assistants and secretaries	6.05 (0.90)	20.40***	Women-dominated
30. General clerical workers	4.65 (1.01)	5.77***	Women-dominated
31. Inquiry clerks and receptionists	5.66 (0.97)	15.38***	Women-dominated
32. Numerical clerks	3.89 (0.94)	-1.07	Gender-neutral
33. Clerical and office support workers	5.01 (1.05)	8.63***	Women-dominated
34. Sales workers	4.24 (0.88)	2.43*	Women-dominated
35. Sales representatives and agents	3.79 (0.79)	-2.40*	Men-dominated
36. Sales assistants and salespersons	4.56 (0.98)	5.14***	Women-dominated
37. Sales support workers	4.35 (0.83)	3.78***	Women-dominated
38. Machinery operators and drivers	1.90 (0.95)	-9.79***	Men-dominated
39. Machine and stationary plant operators	1.91 (0.86)	-1.72***	Men-dominated
40. Mobile plant operators	2.47 (1.07)	-12.78***	Men-dominated
41. Road and rail drivers	2.14 (1.10)	-15.15***	Men-dominated
42. Storepersons	3.89 (1.15)	-0.88	Gender-neutral
43. Laborers	1.86 (0.81)	-23.68***	Men-dominated
44. Cleaners and laundry workers	5.28 (1.15)	9.94***	Women-dominated
45. Construction and mining laborers	1.56 (0.69)	-31.57***	Men-dominated
46. Factory process workers	3.13 (1.07)	-7.30***;	Men-dominated
47. Farm, forestry and garden workers	2.90 (1.11)	-9.06***	Men-dominated
48. Food preparation assistants	4.51 (0.83)	5.55***	Women-dominated

*Note. p* < .05. ** *p* < .01. ***p < .001

To test whether each occupational category was perceived to be men-dominated, women-dominated, or gender-neutral, we conducted one-sample *t*-tests in which we compared the mean of each occupational category to the midpoint of the scale (i.e., 4). Means and standard deviations, along with the results of the one-sample *t*-tests for each occupational category can be found in Table 1. Occupational categories that were (a) significantly lower than the midpoint of the scale (i.e., 4) were coded as men-dominated occupations; (b) significantly higher than the midpoint as women-dominated; and (c) not significantly different from the midpoint as gender-neutral. There were 22 (45.9%) occupations coded as men-dominated, 20 (41.7%) as women-dominated, and 6 (12.5%) as gender-neutral.

Although in our hypothesis statement we only reference men- and women-dominated occupations, some occupations in the existing data set were gender-neutral as noted above. We included them in our analyses to utilize all the data in an exploratory way to shed light on the association between parental leave length and post-leave income in gender-neutral occupations.

### Control Variables

Three covariates were included in our analysis. First, we included the baseline income, which was the financial year gross income reported by participants for the year before the child birth. Second, we included weekly work hours reported in the second year after returning from parental leave (i.e., the year when the work-related outcome variable was measured), given the association between one’s work hours and income. Third, because the archival data included data collected across a long-time span, we included the year in which participants returned to the workplace following their parental leave to control for its effect on salary.

### Analytic Strategy

We used multiple regression analysis to test Hypothesis 1, which posited that women who take a longer (vs. shorter) parental leave experience negative work-related outcomes in men-dominated occupations, whereas such negative work-related outcomes of parental leave are less likely in women-dominated occupations. Specifically, in the regression model predicting post-leave annual income, we included control variables (i.e., baseline income, post-leave weekly work hours, and returning year), parental leave length, two dummy variables of occupational gender type, and interaction terms between parental leave length and occupation gender type. The continuous variables were grand-mean centered. Because occupation gender type is a categorical variable with three categories (i.e., men-dominated, women-dominated, and gender-neutral), we created two dummy-coded variables. Given that our hypothesis focused on the comparison between men- versus women-dominated occupations, we used women-dominated occupation as the reference category. Thus, one dummy variable was constructed with dichotomous coding of “1” referring to men-dominated occupation and “0” referring to non-men-dominated occupation, and the other dummy variable was constructed with dichotomous coding of “1” referring to gender-neutral occupation and “0” referring to non-gender-neutral occupation. Accordingly, two interaction terms were created, one between parental leave length and men-dominated occupation and one between parental leave length and gender-neutral occupation. Given the way the dummy variables were coded, the regression coefficient of the first interaction term reflects how the slope of the association between parental leave length and post-leave income for women working in men-dominated occupations compares to the slope for women working in women-dominated occupations, providing evidence for Hypothesis 1.

Further, given that we coded three types of occupations, we re-ran the regression analysis with gender-neutral occupation as the reference category to gain complete knowledge about the comparisons between the three types of occupations. The results are presented in Table 3.

### Study 1 Results and Discussion

In the dataset used for the current study (*N* = 657), there was no missing value in any of our study variables for hypotheses testing (i.e., parental leave length, occupation gender type, and post-leave annual income). Missing data only existed for control variables (i.e., 68 participants with missing data for post-leave weekly work hours and 160 participants with missing data for baseline annual income). Using Expectation-Maximization algorithm for imputation, listwise deletion, or pairwise deletion resulted in similar results regarding the interaction between parental leave length and occupation gender type, except that when using Expectation-Maximization algorithm, the relationship between parental leave length and post-leave income was significantly stronger for women in men-dominated (vs. gender-neutral) occupations, while this effect was marginally significant when using pairwise deletion and not significant when using listwise deletion. We report regression analysis results with pairwise deletion in the manuscript. Means, standard deviations, and bivariate correlations among the variables are presented in Table 2. Participants’ post-leave income was negatively correlated with parental leave length (*r* = − .10, *p* = .009).

**Table 2:** Study 1: Means, Standard Deviations, and Zero-Order Correlations

Variable	Mean	*S.D*.	1	2	3	4	5	6	7
1. Baseline annual income	33,546.90	29,397.54							
2. Post-leave weekly work hours	27.04	13.10	0.28**						
3. Returning year	2007.42	3.38	0.18**	− 0.03					
4. Parental leave length (in years)	0.94	0.89	− 0.10*	− 0.06	− 0.07				
5. Occupation: Men-dominated vs. others	0.16	0.37	0.14**	0.16**	− 0.02	0.01			
6. Occupation: Women-dominated vs. others	0.70	0.46	− 0.05	− 0.08*	0.04	0.02	− 0.67**		
7. Occupation: Gender-neutral vs. others	0.14	0.35	− 0.08	− 0.06	− 0.04	− 0.04	− 0.17**	− 0.62**	
8. Post-leave annual income	36,760.08	52,715.18	0.45**	0.34**	0.15**	− 0.10**	0.14**	− 0.09*	0.03

*Note.* **p < .01. *p < .05. *N* = 497 for baseline income, *N* = 589 for post-leave weekly work hours, and *N* = 657 for the other variables. Correlations are based on pairwise deletion

**Fig. 1:**
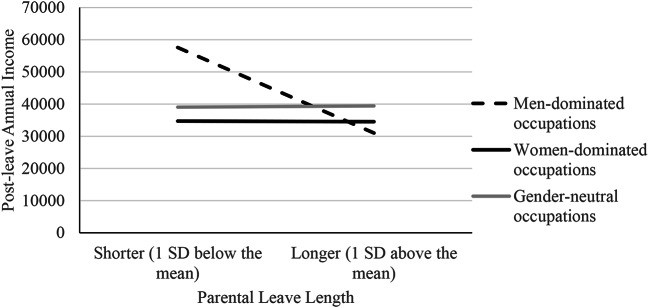
Study 1: Interaction Between Occupation Gender Type and Parental Leave Length in Predicting Post-Leave Annual Income

The results of the multiple regression analysis are presented in Table 3. Overall, 27.1% of the variance in post-leave income was explained by the entire set of variables, *F*(8, 431) = 20.04, *p* < .001. We found a significant interaction between parental leave length and men-dominated occupation on post-leave income (*b* = -13,718.67, *p* = .022), indicating that the relationship between parental leave length and post-leave income was significantly different for those working in men-dominated compared to women-dominated occupations. To facilitate the interpretation of this interaction, we conducted simple slope tests (see Fig. 1). Women working in men-dominated occupations who reported longer (vs. shorter) parental leave reported significantly lower annual income after returning to the workplace (*b* = -13,813.91, *p* = .005). For women working in women-dominated occupations, the simple slope of parental leave length did not significantly predict post-leave income (*b* = -95.24, *p* = .779). Thus, Hypothesis 1 was supported.

**Table 3:** Study 1: Multiple Regression Results for Predicting Post-Leave Annual Income

Variables	*b*	*SE*	*β*
*Female-dominated occupation as the reference category*			
Intercept	34,625.42**	2,586.78	
Baseline income	0.62**	0.08	0.35
Post-leave weekly working hours	921.42**	174.75	0.23
Returning year	1,542.31*	656.95	0.10
Parental leave length (in year)	-95.24	2,763.32	− 0.00
Occupation: Men-dominated vs. others	9,673.02	6,128.50	0.07
Occupation: Gender-neutral vs. others	4,615.35	6,408.87	0.03
Parental leave length x Occupation: Men-dominated vs. others	-13,718.67*	5,988.23	− 0.11
Parental leave length x Occupation: Gender-neutral vs. others	290.72	6,577.01	0.00
*Gender-neutral occupation as the reference category*			
Intercept	39,239.66**	5,869.31	
Baseline income	0.62**	0.08	0.35
Post-leave weekly working hours	921.61**	174.79	0.23
Returning year	1,542.41*	656.95	0.10
Parental leave length (in year)	175.72	5,967.34	0.00
Occupation: Men-dominated vs. others	5,058.29	8,116.77	0.04
Occupation: Women-dominated vs. others	-4,614.34	6,408.83	− 0.04
Parental leave length x Occupation: Men-dominated vs. others	-13,989.58†	8,016.59	− 0.11
Parental leave length x Occupation: Women-dominated vs. others	-266.72	6,577.74	− 0.00
*R*^*2*^	0.271		
*F*	20.04**		

*Note.* **p < .01. *p < .05. †*p* < .10

Further, the interaction between parental leave length and gender-neutral occupation (*b* = 290.72, *p* = .965) was not significant, indicating that the relationship between parental leave length and post-leave income was not significantly different for women in gender-neutral compared to women-dominated occupations. For women working in gender-neutral occupations, the simple slope of parental leave length in predicting post-leave income (*b* = 195.48, *p* = .819) was not significant. Because the outcome variable, post-leave income, was positively skewed, as a robustness check, we re-ran the regression analysis with post-leave income transformed using the square root transformation and the log transformation. The results were largely consistent with the results reported above.

Study 1 provides preliminary support for Hypothesis 1 by showing that the longer the parental leave women in men-dominated occupations take, the lower their reported post-leave income in an archival data set, attesting to its real-world relevance. This reduction in income was not reported by women in women-dominated or gender-neutral occupations. However, one of the main limitations associated with archival data is not being able to establish causality and in particular, an endogeneity problem, that is, a concern that unmeasured variables are driving spurious relationships (Barnes et al., 2018).

## Study 2

The main aim of Study 2 is to replicate the results of Study 1 in a controlled experiment to establish the robustness of the results and causality of parental leave on work-related outcomes (see Hideg et al., 2018, for a similar approach). As such, we manipulate the length of parental leave with one condition presenting a standard length of parental leave, namely 12 months, as Study 1 shows that this was an average length of leave that women in Australia take. The second condition presents a short parental leave, namely one month. We then test Hypothesis 1 again in Study 2 by comparing a 12-month and one-month parental leave. Given that this is an experiment and there is no actual income information, we operationalize a work-related outcome as salary recommendation. By doing so, we sought to constructively replicate our findings from Study 1 using a different methodological approach to test the replicability and robustness of the effects found in Study 1 (see Nosek et al., 2012, for a discussion on the importance of replicability for the advancement of science and accumulating robust and true effects).

### Study 2 Method

#### Participants and Procedure

Participants were 328 workers in Australia with hiring experience recruited using Qualtrics, a third-party organization that recruits participants through their research panels and administers online surveys (we paid approximately $10 USD per participant). We aimed for a sample size of at least 50 per condition (for a total of 200 for 4 conditions) consistent with Simmons et al.’s (2013) recommendation to achieve an adequate power for detecting results (i.e., 80%), and used in previous organizational research (e.g., Hideg & Wilson, 2020). To account for inattentive responses, we planned to recruit 300 participants. Qualtrics originally recruited a total of 550 participants, but 222 participants did not have the required hiring experience. Out of 328 participants with hiring experience, 17 participants did not report a recommended salary, which was the dependent variable, and therefore they were excluded.

As such, our final sample consisted of 311 workers in Australia with hiring experience, including 167 (53.7%) women and 144 (46.3%) men. On average, the participants were 40.59 years old (*SD* = 11.99) and had 6.68 years of hiring experience (*SD* = 7.38). They worked across a range of different occupations (e.g., retail, education, engineering, finance, administration). Two hundred and twenty-eight (73.3%) participants identified as White, 17 (5.5%), as South Asian, 15 (4.8%) as East Asian, nine (2.9%) as Aboriginal/Torres Strait Islander, four (1.3%) as Middle Eastern, three (1%) as multiracial, and one (0.3%) as African (34 or 11% unreported).

In an online survey, participants were informed that the purpose of the study was to examine impressions of job applicants at the beginning of the hiring process when minimal information about an applicant is available. This cover story was adapted from previous research (e.g., Hideg et al., 2018) and its purpose was to provide an immersive and realistic experimental setting (Aguinis & Bradley, 2014). Participants were next informed that they would review an internal job posting, an application for that posting, and they would provide their assessment of the applicant. They were then randomly assigned to read either a job posting for a senior electrical engineer (*men-dominated occupation*) or a senior health educator (*women-dominated occupation*) and the corresponding job application. Next, they were randomly assigned to an internal human resource (HR) document indicating that the applicant took either a 12-month or a one-month parental leave. Thus, we employed a 2 (men vs. women-dominated occupation) by 2 (12-month vs. one-month parental leave) between-subjects design. Finally, participants were asked to recommend the yearly salary the applicant should receive if hired for the position, complete demographic questions, and provided with a debriefing letter.

#### Occupation Gender Type Manipulation

We used senior electrical engineer to represent a men-dominated occupation and senior health educator to represent a women-dominated occupation. We chose these industries because these positions were frequently reported in the archival data from Study 1 and the pilot study described in Study 1 revealed that engineering positions were perceived as typically held by men, and health-industry positions as typically held by women. As noted above, all materials for Study 2 and 3 including the description of these two positions can be found on the OSF website (https://osf.io/ujacn/?view_only=784379d51d5a47e3a8c9dc1c11553cf0). The job advertisement and application were adapted from previous research (Heilman & Okimoto, 2008; Hideg et al., 2018). The job advertisement included the job title (“senior electrical engineer” or “senior health educator”), common tasks associated with the job (e.g., *senior electrical engineer*: inspect project sites to monitor progress and conform to specifications and standards; *senior health educator*: develop, conduct, and coordinate health needs assessment and other public health surveys), and a salary range ($85,000-$135,000).

The common tasks for each position were patterned after information on Occupational Information Network (O*NET). The salary range included average salaries in both fields according to Australian Government sources (Butt et al., 2019). We also asked participants in this study what they thought an average salary for these positions was and found that they were within the given range (senior electrical engineer: *M* = $104,685.55, *SD* = 18,384.71; senior health educator: *M* = $97, 271.23, *SD* = 18, 277.25), although the perceived average salary was higher for a senior electrical engineer compared to health educator, *t*(299) = 3.51, *p* < .001. The job application for both positions portrayed a highly qualified woman applicant with a master’s degree, high performance evaluations, and positive supervisor feedback.

#### Parental Leave Manipulation

The parental leave length manipulation was embedded in an ostensible internal HR document presenting a checklist of the applicant’s leaves of absence in the last two years. The document listed the following options for leaves: long service, parental, personal-sick, and personal-other. In both conditions, only the parental leave was checked off, with either 12 months or one month noted. Although the parental leave length variable was a continuous variable in Study 1, given that we manipulated the length of parental leave to establish causality of parental leave in Study 2, we used two specific lengths of parental leave, one representing longer leave (12 months) and one presenting shorter leave (1 month).

We used 12 months as our longer parental leave condition as this was a practically meaningful length for women and reflects the average length of parental leave in Australia. Specifically, in Study 1 we found that the average time taken by women in Australia was 0.94 years. Thus, the longer parental leave condition presented a standard length of a parental leave in this context. Moreover, past work usually shows negative effects for parental leaves one year or longer, suggesting that this length may be a relatively longer length of leave (Rossin-Slater, 2017). We used a one-month leave to represent a short leave because one month seemed a reasonable minimum time women need to take after giving birth. Namely, an ideal comparison condition would be a woman who did not take a parental leave (i.e., comparing women who took vs. did not take a leave) after giving birth but realistically most women in Australia would take at least some length of parental leave after giving birth. In addition, a one-month parental leave comparison is in line with previous work (e.g., Hideg et al., 2018) and taken by women in professional jobs (e.g., doctors, lawyers). Past research also shows that maternity leaves of three months and less are not associated with negative outcomes (Ruhm, 1998).

In this data collection, we also asked participants (a) what they thought a standard length of parental leave is for women in Australia, and (b) to what degree they perceived a 12-month and a one-month leave to be common in Australia on a 5-point scale (1 = *not common at all*, 3 = *somewhat common*, 5 = *very common*). The average perceived standard length of parental leave for women was 8.12 months (*SD* = 4.96 months). Participants perceived a 12-month leave to be common, but not a one-month leave, as indicated by one-sample *t-*tests showing that the mean for a 12-month leave was higher than the mid-point of the scale (i.e., 3; *M* = 3.58, *SD* = 1.11), *t*(157) = 6.54, *p* < .001, whereas the mean for a one-month parental leave was lower than the mid-point of the scale (*M* = 2.32, *SD* = 1.40), *t*(152) = -5.99, *p* < .001.

#### Dependent Measure and Manipulation Checks

We used an open-ended question to ask participants the yearly salary they would recommend for the applicant if hired for the position (Brescoll et al., 2012). We chose this dependent variable to align with the outcome variable (i.e., post-leave income) in Study 1.

We also included five questions to assess sufficient attention was given to reading the vignette. Specifically, after reading the vignette, we asked participants to verify the (a) gender of the applicant; (b) whether the applicant has taken a leave of absence, and, if they indicated “yes,” we asked them to indicate; (c) the reason for the leave of absence using a multiple choice question with the following options: medical leave, parental leave, or sabbatical leave; (d) length of leave; and (e) position the applicant applied for, using a multiple choice question with the following options: senior marketing associate, senior electrical engineer, or senior health educator. All participants correctly identified the gender of the applicant and that the applicant took a parental leave. Most participants (258, 83%) correctly identified the length of the leave and the position applied for (290, 93%).

We included in our analyses participants who did not correctly identify the length of leave or the position applied for given that for the most part that was the only manipulation question they did not answer correctly suggesting an omission or a temporary distraction from the question rather than a lack of engagement with the study overall. In other words, we did not feel it was justifiable to exclude participants for omission of one manipulation check item given the number of other manipulation checks they correctly answered.

### Study 2 Results and Discussion

**Fig. 2:**
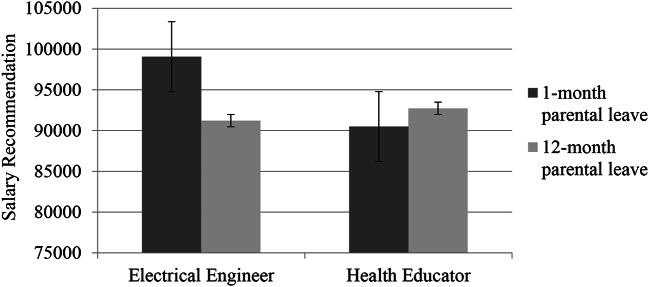
Study 2: Interaction Between Occupation Gender Type and Parental Leave Length in Predicting Women’s Salary Recommendation. *Note*. Error bars represent standard errors (*SE*s)

To test Hypothesis 1, we conducted a 2-way ANOVA to examine the effect of occupation gender type (men- vs. women-dominated) and parental leave length (12-month vs. one-month) on salary recommendation for the woman job applicant. There was no significant main effect of occupation gender type, *F*(1, 307) = 2.26, *p =* .134, ${{\eta}}_{\text{p}}^{\text{2}}$ = 0.01, or parental leave length, *F*(1, 307) = 1.43, *p =*.233, ${{\eta}}_{\text{p}}^{\text{2}}$ = 0.01. However, as expected there was a significant interaction, *F*(1, 307) = 4.60, *p =*.033, ${{\eta}}_{\text{p}}^{\text{2}}$ = 0.02 (see Fig. 2). Follow-up simple slope analyses indicated a lower salary was recommended for the woman applicant applying for an engineering position when she took a 12-month parental leave (*M* = 91,068.75, *SD* = 15,437.15, *n* = 73) compared to a 1-month parental leave (*M* = 99,068.75, *SD* = 21,643.51, *n* = 80), *F*(1, 307) = 5.74, *p* = .017; there was no significant difference in the salary recommendations for the health educator position as a function of taking a 12-month parental leave (*M* = 92,729.65, *SD* = 16,566.54, *n* = 86) compared to a 1-month parental leave (*M* = 90,500.00, *SD* = 27,458.55, *n* = 72), *F*(1, 307) = 0.33, *p* =.563. We also tested for potential effects of participant gender by testing a 3-way interaction between occupation type, parental leave length, and participant gender in predicting recommended salary. This 3-way interaction was not significant, *F*(1, 303) = 0.41, *p =*.521, ${{\eta}}_{\text{p}}^{\text{2}}$ = 0.001.

The results of Study 2 replicated the patterns observed in Study 1 by showing that a woman applying for a men-dominated job who took a 12-month (vs. one-month) leave incurred a penalty of a lower salary recommendation. This penalty was not incurred when the applicant was applying for a women-dominated job. Notably, this pattern emerged even though the woman applicant was portrayed as highly qualified and the qualifications were identical across the conditions. Critically, the 12-month parental leave was typical of the leave taken in Australia, suggesting that women may be penalized for normative leave taking behavior in men-dominated occupations. In Study 2, we thus constructively replicated the finding from archival data in Study 1 by using an experimental design and addressing the issue of endogeneity and causality.

## Study 3

In Study 3, we sought to test Hypothesis 2 and examine whether lower perceptions of women’s agency underlie the negative effects of longer (vs. shorter) parental leaves on work-related outcomes for women in men-dominated occupations. To do so, we conduct another experiment focusing only on engineering as the men-dominated occupation and measured evaluators’ perceptions of the applicant’s agency as an underlying mechanism. In an exploratory fashion, we also measured perceptions of communality as an additional competing underlying mechanism. We also sought to better understand the effect of parental leave length on women’s career progression in Study 3. To do so, we examined the effect of parental leave in the context of a promotion into a managerial position and expanded the breadth of our workplace outcomes by examining perceptions of hireability and leadership effectiveness.

### Study 3 Method

#### Participants and Procedure

We recruited 200 workers and university students in Australia using Prolific, an online crowdsourcing platform designed to recruit participants for scientific research (Peer et al., 2017). We aimed for a larger sample size per condition than in Study 2 to test a mediation model with 100 participants per condition for a total of 200 participants. We originally aimed to recruit only a sample of workers in Australia and thus only opened the survey on Prolific to participants in Australia who indicated they worked (Prolific has this function where surveys could be only opened to certain populations of participants). However, we were unable to recruit an adequate sample size and we thus opened our survey on Prolific to university students in Australia as well. We excluded 30 participants who did not correctly pass three embedded attention checks (Meade & Craig, 2012) and seven participants who did not provide data on our main dependent variable, namely salary recommendation. Thus, our final sample consisted of 163 participants in Australia with 83 (51%) women, 78 (47.9%) men, and two (1.2%) unidentified participants. On average, the participants were 29.75 years old (*SD* = 9.99); and 58 (35.6%) were employed full-time, 65 (39.9%) part-time, 37 (22.7%) were students and not currently employed, and three (1.8%) were with unidentified employment status. The average years of hiring experience was 3.65 (*SD* = 4.64) and out of 37 students, six (16.2%) reported hiring experience. One hundred and twenty-five (76.7%) participants identified as White, ten (6.1%) as Southeast Asian, nine (5.5%) East Asian, six (3.7%) South Asian, one (0.6%) Middle Eastern, and eight (4.9%) multiracial (four or 2.5% unreported).

The procedure for Study 3 resembled the procedure for Study 2 with a few differences. First, all participants were exposed only to the engineering occupation. The position title was slightly modified to fit the position of an engineering manager, as this would allow us to test the effect of parental leave length in a career progression scenario, and the proposed salary range in the job advertisement was shifted accordingly ($90,000-$140,000). The job description was slightly adjusted to reflect an application for a managerial role. Next, as in Study 2, participants were randomly assigned to an ostensible internal HR document indicating that the applicant either took a 12-month or a one-month parental leave. Finally, participants completed a questionnaire assessing their perceptions of the job applicant’s agency, communality, hireability, and leadership effectiveness, provided a salary recommendation, and demographics.

#### Measures and Manipulation Checks

All measures were completed using a 7-point Likert response scale (1 = *strongly disagree*; 7 = *strongly agree*). *Perceptions of agency* were measured with 16 items (e.g., career-oriented, ambitious, competitive; α = 0.83) and *perceptions of communality* were measured with 16 items (e.g., warm, cooperative, supportive; α = 0.92) from Rudman et al. (2012). *Hireability* was measured with a three-item scale (e.g., “I would personally hire the applicant for the job”; α = 0.76) from Rudman and Glick (1999). *Leadership effectiveness* was measured with three items (e.g., “If hired for the position, the applicant would lead a team effectively”; α = 0.93) from Hentschel et al. (2018). *Salary recommendation* was assessed in the same way as in Study 2.

Finally, as an attention check for the manipulation, 160 (98%) participants correctly identified the gender of the applicant; all participants correctly identified that the applicant took a leave of absence, the reason for it (i.e., parental leave), and the length of the leave; and 162 (99%) correctly identified the position. As in Study 2, we included in our analyses participants who did not correctly identify manipulation checks given that for the most part that was the only manipulation question they did not answer correctly suggesting an omission rather than a lack of engagement with the study overall.

### Study 3 Results and Discussion

We first conducted independent samples *t*-tests to examine differences between conditions in perceptions of agency, communality, salary recommendation, hireability, and leadership effectiveness (see Table 4 for means, standard deviations, and zero-order correlations). As expected, we found that women who took a 12-month (vs. one-month) parental leave were perceived lower in agency, *t*(161) = 1.99, *p* = .048, *d* = 0.30, and were rated lower on leadership effectiveness, *t*(161) = 1.98, *p* = .049, *d* = 0.31. There were, however, no significant differences for salary recommendation, *t*(161) = 1.52, *p* = .130, *d* = 0.24, hireability, *t*(161) = 0.94, *p* = .350, *d* = 0.15, or communality, *t*(161) = 0.389, *p* = .698, *d* = 0.07.

**Table 4:** Study 3: Means, Standard Deviations, and Zero-Order Correlations

Variable	One-Month Parental Leave (*n =* 84)	12-Month Parental Leave (*n =* 79)	Overall (*n* = 163)	1	2	3	4
1. Agency	5.42 (0.46)	5.27 (0.53)	5.34 (0.50)				
2. Communality	4.76 (0.62)	4.72 (0.60)	4.74 (0.61)	0.38**			
3. Leadership Effectiveness	5.57 (0.92)	5.28 (0.97)	5.43 (0.95)	0.52**	0.42**		
4. Hireability	5.52 (0.86)	5.39 (0.77)	5.45 (0.81)	0.45**	0.24**	0.59**	
5. Salary Recommendation	107,206 (17,420)	103,455 (13,698)	105,388 (15,790)	0.23**	− 0.08	0.23**	0.25**

*Note.* Columns labelled ‘one-month parental leave,’ ‘12-month parental leave,’ and ‘overall’ present means and standard deviations (standard deviations are in parentheses). For agency, communality, leadership effectiveness, and hireability, the scale ranged from 1 to 7; and the salary recommendation variable presents a dollar value. **p <.01

We also tested for potential effects of participant gender by testing for interactions between parental leave length and participant gender. We found no significant interactions in predicting agency, *F*(1, 157) = 1.45, *p =* .231, ${{\eta}}_{\text{p}}^{\text{2}}$ = 0.01; communality *F*(1, 157) = 0.22, *p =*.641, ${{\eta}}_{\text{p}}^{\text{2}}$ < 0.01; salary recommendation, *F*(1, 157) = 0.01, *p =*.940, ${{\eta}}_{\text{p}}^{\text{2}}$ < 0.01; leadership effectiveness, *F*(1, 157) = 0.11, *p =*.737, ${{\eta}}_{\text{p}}^{\text{2}}$ < 0.01; or hireability, *F*(1, 157) = 0.238, *p =*.63, $\:{\text{η}}_{\text{p}}^{\text{2}}$ < 0.01.

**Fig. 3:**
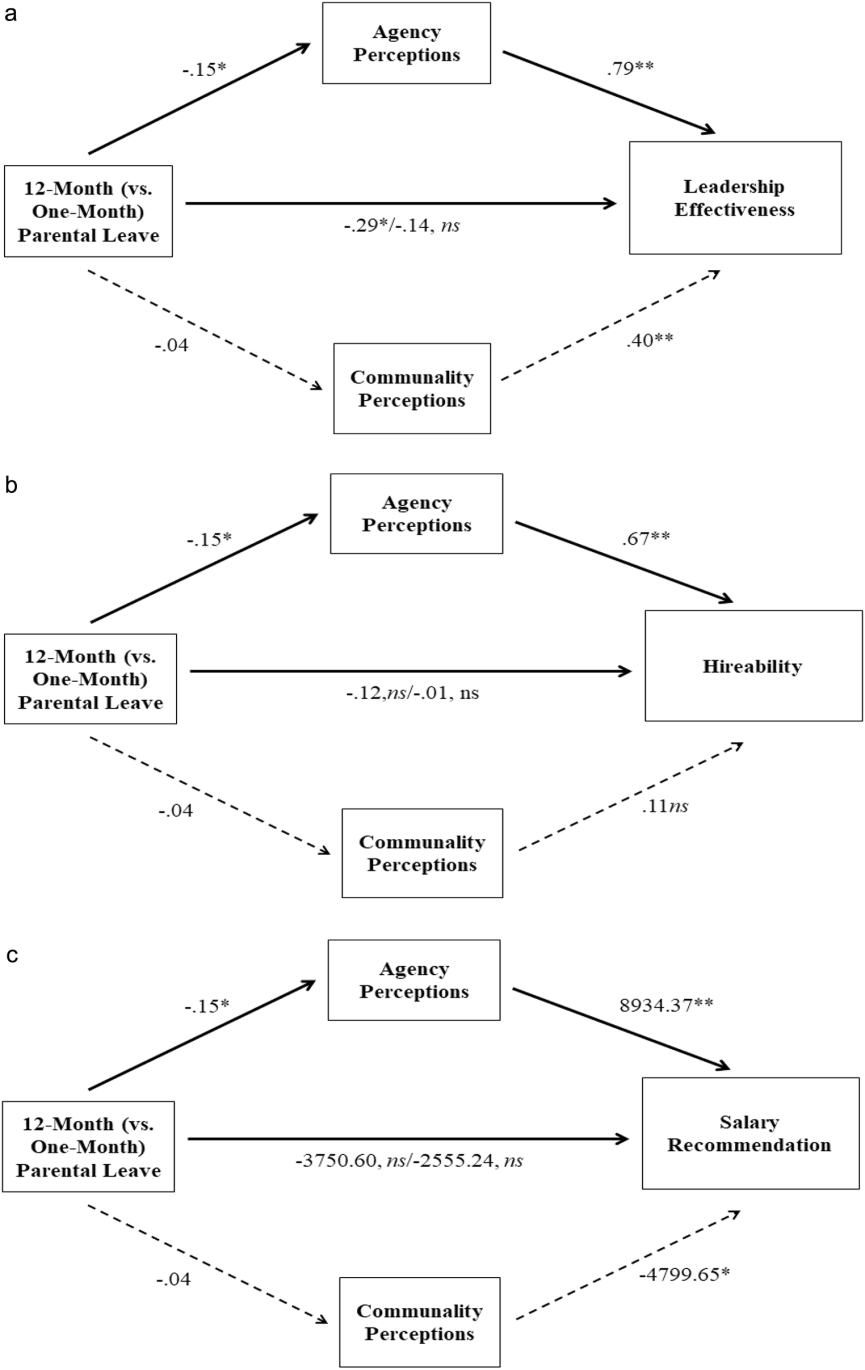
**a** Study 3: Multiple Mediator Model of the Relationship of Parental Leave Length With Leadership Effectiveness via Agency and Communality Perceptions. **b** Study 3: Multiple Mediator Model of the Relationship of Parental Leave Length with Hireability via Agency and Communality Perceptions. **c** Study 3: Multiple Mediator Model of the Relationship of Parental Leave Length with Salary Recommendation via Agency and Communality Perceptions. *Note*. Values are unstandardized coefficients. ns = non-significant. * *p* < .05. ** *p* < .001

We next tested whether agency mediates the effect of longer (vs. shorter) parental leave on the three work-related outcomes (i.e., salary recommendation, hireability, and leadership effectiveness). We also included communality perceptions as a parallel mediator in the model (the conclusions of our analyses remain the same without communality perceptions in the model). As expected, a bootstrapping procedure with 10,000 samples using PROCESS macro (Model 4; Hayes, 2017) showed significant indirect effects of a longer (vs. shorter) parental leave through agency perceptions on salary recommendation (indirect effect = -1,374.24, *SE* = 825.53, 95% confidence interval [CI] [-3,193.07, -24.35]), hireability (indirect effect = − 0.10, *SE* = 0.06, 95% CI [-0.22, − 0.001]), and leadership effectiveness (indirect effect = − 0.12, *SE* = 0.06, 95% CI [-0.25, − 0.001]) (see Fig. 3a-c). Further, as expected, the indirect effect through communality perceptions was not significant given that there was no main effect of parental leave condition on communality perceptions.

In summary, women in men-dominated occupations (i.e., engineering in this study) who took a 12-month (vs. one-month) parental leave were rated lower on leadership effectiveness, but there was no difference for salary recommendation or hireability. However, there were indirect effects of parental leave length on all three outcomes via perceptions of agency, supporting Hypothesis 2. That is, women who took a 12-month (vs. one-month) parental leave were perceived lower in agency, which in turn was related to lower leadership effectiveness, lower salary recommendation, and lower hireability.

### Exploratory Analyses

Recent work suggests that agency perceptions can be broken down into different dimensions, which may provide more nuanced and precise insights into underlying gender stereotypes (Hentschel et al., 2019; Ma et al., 2022; Rosette et al., 2016). While we used a common measure for assessing overall agency perceptions, some of the items tapped into competence perceptions, which are deemed to be a different dimension of agency compared to more traditional views of agency, which tap into assertiveness and ambition. This is relevant to our work as we theorize that perceptions of ambition and career dedication, which are hallmarks of men-dominated occupations and not perceptions of competence, underlie the negative effects of longer parental leaves on women’s work-related outcomes.

To examine whether items relating to traditional agency, and not items relating to competence, mediate our effects, we created two different composite scores based on our 16-item agency measure: an 8-item *agency score* (i.e., career oriented, aggressive, assertive, ambitious, self-starter, high self-esteem, persuasive, and competitive; α = 0.70) and an 8-item *competence score* (i.e., competent, leadership ability, business sense, intelligent, hard working, analytical, works well under pressure, and independent; α = 0.81). In line with our main analyses, we conducted independent sample *t*-tests to examine differences in perceptions of agency and competence. We found that women who took a 12-month (*M* = 5.01, *SD* = 0.57) compared to a one-month (*M* = 5.20, *SD* = 0.53) parental leave were perceived as lower in traditional perceptions of agency, *t*(161) = 2.17, *p* = .031, *d* = 0.34. There were no differences in perceptions of competence (12-month parental leave: *M* = 5.69, *SD* = 0.59; one-month parental leave: *M* = 5.83, *SD* = 0.51), *t*(161) = 1.55, *p* = .124, *d* = 0.24.

Given that there was a difference between the two parental leave conditions only on traditional agency perceptions (and not on competence), we conducted our mediation analyses in which we used this new 8-item agency score instead of the overall 16-item agency score. In line with our main analyses, we included communality perceptions as an additional mediator, and used the same statistical procedures. As expected, there were significant indirect effects of a 12-month (vs. one-month) parental leave through the 8-item agency score on salary recommendation (indirect effect = − 0.1201.05, *SE* = 750.79, 95% CI [-2916.73, -49.19]), hireability (indirect effect = − 0.07, *SE* = 0.04, 95% CI [-0.16, − 0.01]), and leadership effectiveness (indirect effect = − 0.10, *SE* = 0.05, 95% CI [-0.21, − 0.01]). As such, these additional analyses show that lower traditional views of agency, such as ambition, assertiveness, and dedication to career, and *not* perceptions of competence, mediate the negative effect of a 12-month (vs. one-month) parental leave on work-related outcomes for women in men-dominated occupations.

## General Discussion

Using a mixed-method approach consisting of an archival data set and two experiments with samples of workers and students from Australia, we examined the impact of parental leave length on work-related outcomes for women in men-dominated occupations. Using archival data, we found that the longer a woman’s parental leave, the lower their reported annual income in men-dominated occupations. However, such negative effects of longer parental leaves were not observed for women working in women-dominated and gender-neutral occupations. We replicated and extended our findings in two experiments where we found that a standard length of parental leave (12 months), compared to a shorter leave (one month), had a negative effect on work-related outcomes, including lower salary recommendation, lower perceived hireability, and lower perceived leadership effectiveness in a men-dominated, but not a women-dominated, occupation.

We further found that lower perceptions of women’s agency, a trait which men-dominated occupations place a premium on, accounts for the negative relationship between parental leave length and work-related outcomes; whereas perceptions of communality, a trait on which men-dominated occupations do not place a premium, did not mediate these associations. Finally, we did not find any gender differences in Study 2 and 3 suggesting that both men and women evaluators, including those with hiring experience, engage in similar evaluations of women’s agency in men-dominated occupations. This is in line with past research suggesting that subtle prejudices towards women at work could be enacted by both men and women (Hideg & Ferris, 2016; Hideg & Krstic, 2021).

## Theoretical Contributions

Our work makes important contributions to several literatures. First, our work contributes to the literature on parental leave and women’s careers. Past research has generally documented negative effects of parental leave on women’s careers (e.g., Evertsson, 2016; Evertsson & Duvander, 2011; Olivetti & Petrongolo, 2017). Yet, this past work has not considered the effect of occupation gender type in escalating (or potentially ameliorating) the negative effects of parental leave for women’s careers. This omission is significant given that women are underrepresented in traditionally higher paying and prestigious positions which are typically dominated by men. Yet these occupations may accentuate traditional gender stereotypes, bringing on unintended negative effects of parental leave for women that are not generally observed in women-dominated occupations. This suggests it is not always the parental leave per se that influences whether women incur penalties for this behavior, but the context in which it is being taken. Thus, our work identifies a critical boundary condition of previously demonstrated negative effects of parental leave.

Second, by providing evidence that lower perceptions of agency underlie the negative career effects of longer parental leave, our findings suggest that higher agency perceptions ascribed to women who successfully enter men-dominated fields are fragile (Bosak et al., 2012). One communal event (e.g., taking a longer parental leave) seems to be enough to reduce women’s perceived agency in the fields where they are underrepresented the most. Moreover, past work has mostly examined archival data sets precluding examination of mechanisms because such data sets frequently do not have data collected on underlying psychological mechanisms such as perceived stereotypes. One of the key suggested mechanisms in this past work has been human capital depreciation, namely a decline of skills due to job-related knowledge or skills not being used with longer leaves (Evertsson, 2016). Yet, our work shows that even when there is no sign of human capital loss and there is evidence of high performance, women who take longer leaves are still penalized.

Our work also contributes to the literature on women in men-dominated fields (Ceci & Williams, 2011; Cech & Blair-Loy, 2019; Shauman, 2017). It shows that a popular solution of offering longer parental leaves to address underrepresentation of women in men-dominated occupations may unintentionally backfire if traditional gender and sexist stereotypes within the organizational culture are not simultaneously addressed. Critically, we show this negative effect for a standard length of parental leave (i.e., 12 months), which is the average length taken by women in Australia. As such, an otherwise normative behavior of taking a 12-month parental leave, may not be perceived as normative in an organizational structure where a gendered trait of agency is valued. Without addressing these structural issues, real changes in the gender representation gap are unlikely.

Finally, our work contributes to the literature on gender stereotyping (Eagly & Wood, 2012; Koenig & Eagly, 2014) by integrating micro (gender stereotypes) and macro (occupation gender type) factors in understanding consequences of parental leave for women’s careers. We show how an organizational structure dominated by men reinforces and amplifies gender stereotypes. Past research on the effects of parental leave on women’s careers (e.g., Hideg et al., 2018) has shown that taking parental leave invokes traditional gender stereotypes, which undermine women’s careers. Critically, this past work has conceptualized stereotyping as an individual difference (i.e., coming from and being reinforced by individuals). Our work demonstrates a key role for organizational structures (regardless of individual endorsement of gender stereotypes) in reproducing and reinforcing stereotypes and gender inequity.

Our work also contributes to a shift in the natural and social sciences by focusing specifically on women’s experiences and focusing on perceptions of women as focal persons of interest in a scientific inquiry. This is in contrast to a widespread men bias in scientific research, which traditionally has not been very inclusive and has treated men and their experiences as the default (Crasnow, 2020; Hideg, Hancock, & Shen, 2023; Perez, 2019). Importantly, we wish to clarify that our work does not suggest that opportunities for longer parental leave are ultimately detrimental to women’s careers and should not be offered or taken. On the contrary, we suggest that offering such leave is a baseline minimum to attract women to these occupations, but we also highlight that it is not enough just to offer such policies.

Importantly, more substantial support for women’s careers as well as significant cultural and normative changes are needed to help them succeed (Zhang, 2020). Namely, the work on masculinity contest cultures (Berdahl et al., 2018; Glick et al., 2018) suggests that men-dominated occupations have toxic cultures exemplified with overwork and cutthroat competition. It appears that offering parental leave benefits on its own will not support women’s careers and success in such cultures. Rather, organizations may need more fundamental changes in their culture and leadership style (Hideg, Krstic, et al., 2023). Although women may be disproportionately affected by work-life issues and masculine cultures, such cultures and long work hours, where it is difficult to combine work and life domains, hurt men as well (Padavic et al., 2020; Reid, 2015). The negative effects of longer parental leave incurred by women in men-dominated occupations may thus be a signal of the problematic sexist culture embedded within many of these occupations.

### Limitations and Future Research Directions

Our work also has limitations. First, we did not observe direct effects of parental leave length on all outcomes in Study 3. We observed direct effects on agency and leadership effectiveness, but not on salary recommendation or hireability, although the mean comparisons were in the predicted direction (lower in the 12-month vs. one-month parental leave condition). Although we do not have a definitive answer for why this happened, some possibilities include that the effects could have been diluted since some of our participants were students who have less experience with hiring (especially hiring for a management position as in Study 3), less understanding of parental leave in general, and/or tend to be more liberal in general. In addition, we included in our analyses people who did not answer all manipulation checks correctly (although they did answer the majority correctly), which could have added some noise in our results. However, critically, we still observed an indirect effect on salary recommendation and hireability via agency perceptions.

Next, HILDA data analyzed in Study 1 used a binary view of gender omitting other gender identities and did not collect information on other intersecting identities (e.g., race) thus limiting our findings to ‘default’ women, which tend to be White, cisgender, and heterosexual (e.g., Cross et al., 2021). Similarly, in Study 2 and 3, there was no explicit information presented on women’s other identities in our materials. More research is thus needed on other gender identities and intersection of gender with other marginalized identities. Further, our studies were conducted in the context of Australian parental leave taking limiting our conclusion to this cultural context and to other developed countries with similar maternity leave policies (e.g., Canada, Western Europe). Future work should examine the effects of parental leaves on women’s careers in men-dominated occupations in non-Western countries and economies.

We also note that the HILDA data used in Study 1 were collected between 2001 and 2013, which may invoke the question of whether these data are still relevant. We believe our findings based on the data are still relevant today for several reasons. First, gender stereotypes are deeply embedded in society and resistant to change. While research has shown that belief of male advantage in competence has weakened, the traditional perceptions of agency have been strongly associated with men and remained unchanged (Eagly et al., 2020). Indeed, Charlesworth and Banaji (2022) reported that the male-career/female-family stereotype could take at least 134 years from 2018 to reach neutrality, even though they observed a weakening trend of this stereotype between 2007 and 2016. As such, there is little change over time in the underlying mechanism that explains the observed effect of parental leave length. Second, there has not been any drastic change in the gender composition in men-dominated occupations (Borland, 2022) or in the norm of parental leaves in Australia over the past decade (Baird et al., 2021).

Further, in Study 1 we coded occupations as men-dominated, women-dominated, and gender balanced based on a pilot study where participants rated the degree to which they perceived occupations to be typically held by women or men. This may invoke a question to what degree these perceptions are accurate. While not perfect, Cejka and Eagly (1999) found that participants’ estimates of gender distribution across occupation were significantly correlated with the distributions reported in census data. Moreover, research shows that occupations used in Study 2 and 3, namely engineering and healthcare are mostly (70% and more) occupied by men and women respectively (e.g., Boreland, 2022), further attesting to the validity of our manipulations.

Our work also lays the groundwork for potential future research. One avenue is to examine additional and potentially related underlying mechanisms in addition to traditional agency perceptions identified in this paper. As discussed above, organizational cultures such as masculinity contest cultures (Berdahl et al., 2018), which are prominent in men-dominated occupations, may be an important factor to be examined. Such cultures value assertiveness, ambitions, and competitiveness, and any signal that may bring these traits into question (such as parental leaves) stands to undermine employees’ careers. We encourage future work on underlying organizational cultures in men-dominated occupations that may undermine women’s success, especially for those with a post parental leave experience.

This also brings into question whether men in men-dominated occupations who take parental leaves are penalized. Some past work suggests that men who take flexible work arrangements, including family related leaves, are penalized at work (Coltrane et al., 2013; Rudman & Mescher, 2013) because they are perceived as deficient in agency. By contrast, recent work suggests that men in professional jobs who take parental leaves may incur positive career outcomes due to increased perceptions of communality, although their agency is preserved (Fleischmann & Sieverding, 2015; Krstic & Hideg, 2019). Thus, there is a possibility that men’s visible engagement with family-related matters at work, including taking parental leaves, may help shift some of the negative stereotypes and consequences associated with parental leaves. Future work should examine this possibility in both men- and women-dominated occupations.

### Practice Implications

Our work suggests that, if not managed effectively, longer parental leave can have unintended negative consequences for women thereby further reinforcing gender inequality. One important practical implication for organizations and managers stemming from this work is that the implementation of such family-friendly benefits and practices needs to be accompanied by attention to how employees and managers interpret these policies (Rothbard et al., 2005) and providing additional supports for women’s careers, ensuring their long-term success. At minimum, managers and other decision-makers need to be aware that women who take parental leave may be evaluated less favorably simply due to gender stereotypes and need to look for policies and practices to counter such stereotypes.

The responsibility of addressing this issue, however, should not solely rest on individual managers. Rather, organizations should be implementing processes and practices that accompany family-friendly benefits such as parental leave to ensure that their uptake does not have unintentional effects on women’s support and growth in the workplace. Some potential initiatives include incorporating information on gender biases ensuing from taking parental leave in training sessions on diversity, inclusion, and bias; ensuring that every manager undergoes such training; and offering support (e.g., coaching) with returns from parental leaves and transitioning back to work. It is also important for men in men-dominated organizations to be allies and aware of these issues when supporting women since they tend to have more visibility and decision-making power in these environments (e.g., Hardacre & Subasic, 2018). Finally, organizations can also provide opportunities for women to reaffirm their agency through stretch assignments (i.e., an assignment going beyond one’s current level and skills) and high-performance projects.

Critically, our work points to the need for deeper cultural and normative changes that need to happen in men-dominated occupations. Namely, organizational cultures in these occupations may be feeding into the necessity of strong agentic traits even when competence and high performance have been demonstrated. That is, our work suggests that in occupations where a premium is not placed on agentic traits, such as in women-dominated occupations, negative effects of parental leave are less likely to occur. As such, a key longer-term implication for organizations is to create and cultivate leaders, organizational cultures, and norms that place less emphasis and value on traditional agentic traits and potentially focus more on communal traits and benefits that such traits may bring to their job or occupation (Danbold & Bendersky, 2020). This may ultimately lead to a more inclusive, balanced, and healthier workplace. As previous research shows, cultures of long hours and lack of flexibility negatively affect both women and men (Padavic et al., 2020). This research may support decision-makers in men-dominated occupations to address organizational cultures which not only undermine women, but more broadly undermine the well-being of all employees.

## Conclusion

Across one archival and two experimental studies in the Australian context, we found support for our hypotheses that women in men-dominated occupations may incur career penalties after taking a standard longer (vs. shorter) leave due to lowered perceptions of agency on which men-dominated occupations put a premium. By contrast, we did not find these negative effects in women-dominated occupations. By integrating micro and macro perspectives on gendered work-related outcomes in organizations, our results highlight that whether taking much needed parental leave undermines women’s careers is determined by the effect that leave has on perceptions of women’s agency, which is communicated through the gendered structure of the occupational environment in which women work. Without addressing these structural barriers in men-dominated occupations, gender equity will remain elusive.

## Data Availability

In Study 1 we used an archival data set, the Household, Income and Labour Dynamics in Australia (HILDA) Survey, published by the Melbourne Institute. The access and use of this data set are managed and regulated by the Melbourne Institute (please see https://melbourneinstitute.unimelb.edu.au/hilda) and thus we do not have permission to share this data set. Study 2 and 3 were experiments and were reviewed and approved by an Institutional Review Board. The protocol number was 5142 and the title of the project was “Evaluating job applicants.” Data from Study 2 and 3 cannot be shared publicly because we do not have consent from our participants to do so; however, data are available on an individual basis and can be requested from the first author.
